# Efficacy of Bilateral Cochlear Implantation in Pediatric and Adult Patients with Profound Sensorineural Hearing Loss: A Retrospective Analysis in a Developing European Country

**DOI:** 10.3390/jcm12082948

**Published:** 2023-04-18

**Authors:** Claudia Raluca Balasa Virzob, Marioara Poenaru, Raluca Morar, Ioana Delia Horhat, Nicolae Constantin Balica, Reshmanth Prathipati, Radu Dumitru Moleriu, Ana-Olivia Toma, Iulius Juganaru, Vlad Bloanca, Gratiana Nicoleta Chicin, Roxana Manuela Fericean, Eugenia Maria Domuta, Mircea Iurciuc, Stela Iurciuc

**Affiliations:** 1 Department of Ear-Nose-Throat, Faculty of General Medicine, “Victor Babes” University of Medicine and Pharmacy Timisoara, Eftimie Murgu Square 2, 300041 Timisoara, Romania; virzob.claudia@umft.ro (C.R.B.V.); poenaru.marioara@umft.ro (M.P.); raluca.morar@umft.ro (R.M.); horhat.ioana@umft.ro (I.D.H.); balica@umft.ro (N.C.B.); 2 Santiram Medical College and General Hospital, Faculty of Genenral Medicine, Nandyala 518001, Andhra Pradesh, India; 3 Mathematics Department, Faculty of Mathematics and Computer Science, West University Timisoara, 4th Vasile Parvan, 300223 Timisoara, Romania; radu.moleriu@e-uvt.ro; 4 Discipline of Dermatology, Faculty of General Medicine, “Victor Babes” University of Medicine and Pharmacy Timisoara, Eftimie Murgu Square 2, 300041 Timisoara, Romania; toma.olivia@umft.ro; 5 Department of Pediatrics, Faculty of General Medicine, “Victor Babes” University of Medicine and Pharmacy, 300041 Timisoara, Romania; juganaru.iulius@umft.ro; 6 Department of Plastic Surgery, Faculty of General Medicine, “Victor Babes” University of Medicine and Pharmacy Timisoara, Eftimie Murgu Square 2, 300041 Timisoara, Romania; 7 Faculty of General Medicine, “Vasile Goldis” Western University of Arad, Bulevardul Revolutiei 94, 310025 Arad, Romania; 8 National Institute of Public Health, Strada Doctor Leonte Anastasievici 1–3, 050463 Bucuresti, Romania; 9 Doctoral School, Faculty of General Medicine, “Victor Babes” University of Medicine and Pharmacy Timisoara, Eftimie Murgu Square 2, 300041 Timisoara, Romania; 10 Surgery Department, Faculty of Medicine and Pharmacy, University of Oradea, Piata 1 Decembrie 10, 410073 Oradea, Romania; 11 Department of Cardiology, Faculty of General Medicine, “Victor Babes” University of Medicine and Pharmacy Timisoara, Eftimie Murgu Square 2, 300041 Timisoara, Romania; 12 Research Center of the Institute of Cardiovascular Diseases Timisoara, Faculty of General Medicine, “Victor Babes” University of Medicine and Pharmacy Timisoara, Eftimie Murgu Square 2, 300041 Timisoara, Romania

**Keywords:** cochlear implants, hearing loss, audiology, correction of hearing impairment

## Abstract

This retrospective study aimed to evaluate the outcomes of bilateral cochlear implantation in patients with severe-to-profound sensorineural hearing loss at the Timisoara Municipal Emergency Clinical Hospital ENT Clinic. The study involved 77 participants, divided into four groups based on their hearing loss characteristics and implantation history. Assessments were conducted pre- and post-implantation, focusing on speech perception, speech production, and reading achievement. Standard surgical procedures were performed, and participants were provided with a comprehensive rehabilitation program involving auditory training and communication therapy. The variables considered for analysis included demographic factors, implantation period, and quality of life assessment, with no statistically significant differences pre-implantation between the four study groups. Results revealed significant improvements in speech perception, speech production, and reading achievement after cochlear implantation. In adult patients, speech perception scores increased from 21.3% to 73.4% for WIPI and from 22.7% to 68.4% for HINT after 12 months of rehabilitation. Speech production scores improved from 33.5% to 76.8% and reading achievement scores increased from 76.2 to 106.3. Moreover, there was a significant improvement in patients’ quality of life following cochlear implantation, with mean scores increasing from 2.0 to 4.2. Although it is known that bilateral cochlear implantation significantly improves speech perception, speech production, reading achievement, and quality of life in patients with severe-to-profound sensorineural hearing loss, this is the first study of its kind from Romania. Further research is warranted to optimize patient selection and rehabilitation strategies to maximize outcomes and determine better policies towards funding and access of cochlear implants for a wider range of patients in need.

## 1. Introduction

Hearing loss is a pervasive and under-addressed global health issue that can result in social isolation, depression, loss of autonomy, and neuropsychological dysfunction in affected individuals. Consequently, individuals with hearing loss often face barriers to workforce integration, leading to reduced economic security and increased utilization of healthcare resources [1,2]. Auditory deficiencies are recognized as a significant impediment to the communication process and are identified as a primary etiological factor in language development and speech impairment [3]. Cochlear implantation has emerged as the standard intervention for children with severe to profound hearing loss and has experienced considerable advancements in recent years, aided by novel technologies that have enhanced the quality of life for numerous children and adults [4,5,6,7].

Cochlear implants are prosthetic devices that enable individuals with severe-to-profound hearing loss to regain their ability to perceive sounds [8,9,10]. The outcomes of cochlear implantation can vary across individuals, with factors such as age at implantation and duration of implant usage playing a crucial role in language development, communication skills, and speech comprehension [11,12,13].

During rehabilitation, it is essential to engage in discussions with children and their parents about potential issues to optimize care services for cochlear implant recipients [14,15,16,17,18,19]. Research examining the benefits of bilateral cochlear implantation has revealed improved speech and auditory perception of noise for children receiving a second implant compared to those with a single implant and bilateral sensorineural hearing loss [20,21]. The time interval between implants does not impact the outcomes of the second implant, but the duration of bilateral cochlear implant use is vital for efficiency, adaptation, and audiological result progression, as it promotes the preservation of the corresponding auditory pathway [22,23,24,25,26].

The cost-effectiveness of cochlear implants must be considered; in the United States, bilateral implantation in children and adults is deemed cost-effective, while pediatric sequential, adult bilateral, elderly, and long-term deaf implantations are viewed as less so [27,28]. Approximately 300,000 individuals worldwide have cochlear implants, and the German Federal Statistical Office reported more than 3700 cochlear implant recipients from birth to 95 years old in 2015 [29], although data from the less developed European countries is scarce regarding the population affected by hearing loss and the number of implants performed. One study reported that data on cochlear implant recipients were collected from 15 European countries, utilizing various sources such as government records, cochlear implant teams, and ENT reports [30]. However, caution should be exercised when comparing data from different sources and considering the absence of data from almost one-third of European countries.

It is concerning that there is such limited information on cochlear implant recipients, and obtaining such information proves challenging. The WHO also acknowledges the lack of valid data on hearing care, urging member states to collect high-quality, population-based data on ENT diseases and hearing loss to develop evidence-based strategies and policies. Therefore, it was necessary to investigate and report the juvenile and adult patients who underwent a bilateral cochlear transplant in Western Romania. The primary objective of the current research was to study the impact of the cochlear implant in children and adults with bilateral hearing loss on speech perception, speech production, and reading. The secondary objective of the study was to assess the quality of life after implantation and vocal and auditory rehabilitation.

## 2. Materials and Methods

### 2.1. Study Design and Variables

The current study followed a retrospective design according to the STROBE guidelines [31] and focused on patients who underwent bilateral cochlear implant procedures at the Timisoara Municipal Emergency Clinical Hospital ENT Clinic, Cochlear Implant Department, over a five-year span (2016–2020). Approval for the study was granted by the Ethics Committee of the Timisoara Municipal Emergency Clinical Hospital (No. I-28406/28 October 2022), ensuring that all patients involved provided their written informed consent.

All included patients underwent bilateral cochlear implantation under standard surgical procedures. The participants were implanted with different cochlear devices based on availability and funding at the time of intervention. The clinic offered a comprehensive vocal and auditory rehabilitation program for implant recipients during the first month after implantation, followed by several appointments over one year. The follow-up period and patients’ assessment encompassed one appointment for the initiation of the implant and an additional three appointments dedicated to follow-up at three months, six months, and 12 months. The program involved simultaneous auditory training and communication therapy, both of which were directed by goals identified by the recipient prior to implantation. Auditory training was carried out solely through auditory means and initially emphasized phonetic and phrase-level materials. Recipients were taught to utilize available linguistic cues to bridge perceptual gaps during conversations. The objective of communication therapy was to augment recipients’ awareness and management of communication barriers across various situations.

The study conducted three assessments: speech perception, speech production, and reading achievement. Speech perception was evaluated using the Word Intelligibility by Picture Identification test (WIPI) [32] and the Phonetically Balanced Kindergarten Word List (PBK) for young children [33], while the Hearing in Noise Test (HINT) was utilized for adults to assess open-set sentence perception [34]. Speech production skills for adults were assessed through a short-long sentence repetition task, where participants imitated speech and sign models presented by a speech-language pathologist. Phonetic transcriptions were scored based on the percentage of correct phonemes produced. Reading comprehension ability was measured using the Passage Comprehension subtest from the Woodcock Reading Mastery Tests–Revised [35], which assessed participants’ ability to comprehend short passages and supply missing words. Participants could provide answers through sign, voice, and sign or voice only.

The variables considered for analysis comprised: the place of origin, gender, age, implantation age, implantation period, development of otitis after cochlear implantation procedure, speech perception scores, speech production scores, reading achievement scores, and quality of life assessment using the mental domain of the World Health Organization Quality of Life—Brief version survey (before and after the procedure) [36]. Quality of life reflects the adults’ and the child’s family’s view about the impact of treatment.

### 2.2. Patient Eligibility and Study Groups

Data were collected from the hospital database comprising digital and paper records of all admitted patients. Eligible patients admitted to our audiology clinic underwent a thorough screening process to confirm the diagnosis of profound sensorineural hearing loss and ensure they met the criteria for cochlear implantation. To be included in the study, both children and adults needed to meet specific criteria. They must have experienced a bilateral profound sensorineural hearing loss of 85 dB or greater, with little to no speech recognition benefits from hearing aids, or they require the replacement of an external processor due to advanced physical wear. Another inclusion criterion was the existence of a pre-and post-implant assessment of speech perception. Baseline measures of speech perception were collected using closed-set and open-set speech perception tests. Participants also completed a self-report survey to assess their pre-implant expectations and satisfaction with their current hearing aids. Exclusion criteria were also established: (1) patients with recent or current ear infections or other acute illnesses; (2) having incomplete investigations and incomplete personal records; (3) patients over the age of 65 years; (4) lack of consent; (5) patients with unilateral sensorineural hearing loss; and (6) patients with syndromes and/or neurological or global development changes were not considered for inclusion in the current study.

**Figure 1:**
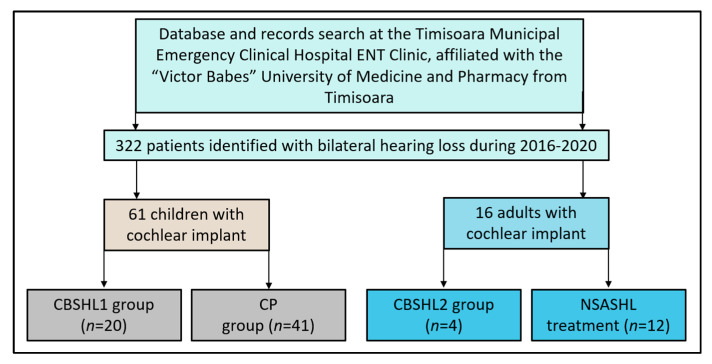
Study flowchart; CBSHL1—patients with congenital bilateral sensorineural hearing loss receiving their first implant; NSASHL—patients with non-congenital severe acquired bilateral sensorineural hearing loss; CBSHL2—patients with congenital bilateral sensorineural hearing loss; CP—previously implanted patients who were hospitalized for the change of processor (control group).

To effectively analyze the data, the patient cohort was divided into four distinct groups, as described in Figure 1. Group 1 consisted of patients receiving their first cochlear implant due to congenital bilateral sensorineural hearing loss (CBSHL1). Group 2 comprised patients with non-congenital severe acquired sensorineural hearing loss (NSASHL). Group 3 included patients receiving a second cochlear implant for congenital bilateral sensorineural hearing loss (CBSHL2). Lastly, Group 4 was considered as the control and consisted of patients with bilateral sensorineural hearing loss who were hospitalized for the purpose of changing their cochlear implant processor (CP). This comprehensive grouping structure allowed for a thorough investigation of various patient experiences and outcomes related to cochlear implantation.

### 2.3. Statistical Analysis

Statistical analysis was conducted using JASPv16.3 and Microsoft Excel 365 software. Initially, a descriptive analysis was performed to characterize variables, calculating central tendency and dispersion parameters for numerical variables and representing the results using boxplots. Median, mode, minimum, maximum, and range values were calculated for ordinal variables. Central tendency and dispersion parameters were calculated for numerical variables, and data distribution was tested based on the four subgroups. Median and interquartile range (IQR) data were represented using boxplots. Frequency tables were generated for qualitative, dichotomous, and ordinal variables, with pie charts used to display the outcome for the latter two.

The Shapiro-Wilk test was applied to test the distribution of numerical variables, revealing non-normal distribution (*p* < 0.05). To assess observed differences, the Mann-Whitney U test was employed for two groups, the Kruskal-Wallis test for more than two groups, and the Wilcoxon signed-rank test for ordinal variables. Correlations between ordinal variables were evaluated using a correlation model, with the Spearman parameter determining correlation strength. A risk analysis was conducted, calculating odds ratios (OR) and 95% confidence intervals, with the chi-square test applied to determine statistical significance. The significance level was set at α = 0.05.

## 3. Results

### 3.1. Patients’ Background

Table 1 presents the general characteristics of the study groups, which include patients with congenital bilateral sensorineural hearing loss receiving their first implant (CBSHL1, *n* = 20), patients with non-congenital severe acquired bilateral sensorineural hearing loss (NSASHL, *n* = 12), patients with congenital bilateral sensorineural hearing loss (CBSHL2, *n* = 4), and previously implanted patients who were hospitalized for the change of processor (CP, *n* = 41). The variables analyzed include the place of origin, gender, age, implantation age, and implantation period. In terms of place of origin, the majority of patients in all groups resided in urban areas, with 65.0% of CBSHL1 patients, 75.0% of CBSHL2 patients, 75.0% of NSASHL patients, and 61.0% of CP patients. The remaining patients in each group were from rural areas, with no significant differences among the study groups in terms of place of origin (*p*-value = 0.854).

Regarding gender distribution, there were more male patients in the CBSHL1 and CBSHL2 groups, with 65.0% and 50.0%, respectively, while the NSASHL and CP groups had more female patients, with 66.7% and 58.5%, respectively. Therefore, there was no significant difference among the study groups regarding gender distribution (*p*-value = 0.334). The mean age of the patients varied significantly among the groups, with the CBSHL1 group having a mean age of 2.1 years, CBSHL2 at 37.3 years, NSASHL at 17.8 years, and CP at 1.9 years. The *p*-value of 0.003 indicates a significant difference in the mean age of the patients across the study groups.

**Figure 2:**
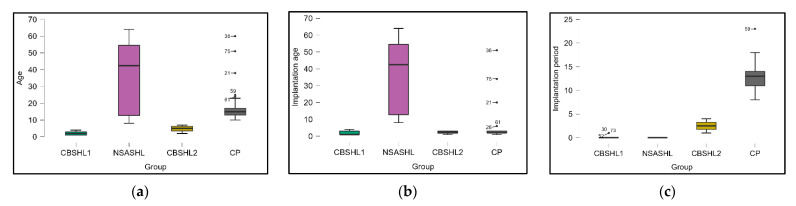
The data dynamics within the four studied groups $N_{CBSHL1}=20;N_{NSASHL}=12;N_{CBSHL2}=4;N_{CB}=41$: (**a**) the age of patients; (**b**) the implantation age; (**c**) the implantation period; CBSHL1—patients with congenital bilateral sensorineural hearing loss receiving their first implant; NSASHL—patients with non-congenital severe acquired bilateral sensorineural hearing loss; CBSHL2—patients with congenital bilateral sensorineural hearing loss; CP—previously implanted patients who were hospitalized for the change of processor (control group).

Implantation age also demonstrated substantial differences between the groups, as described in Figure 2. The mean implantation age for the CBSHL1 group was 1.95 years, 37.33 years for CBSHL2, 2.25 years for NSASHL, and 4.73 years for CP. The *p*-value of <0.001 reveals a highly significant difference in the mean implantation age among the study groups. Lastly, the implantation period, which refers to the duration between the onset of hearing loss and the cochlear implantation, showed significant variation across the groups. The mean implantation period for the CBSHL1 group was 0.15 years, 0 years for CBSHL2, 2.5 years for NSASHL, and 13.02 years for CP. Therefore, there was a highly significant difference in the mean implantation period among the study groups (*p*-value < 0.001). It was also observed that a significantly higher proportion of children with congenital bilateral sensorineural hearing loss developed otitis after the first cochlear implant compared with their adult counterparts (75.0% vs. 25.0%, *p*-value = 0.001).

A Mann-Whitney test was conducted to evaluate differences in age, implantation age, and implantation period among patients based on their gender, environment, main procedure, and the presence/absence of associated diseases. The results show that there were no statistically significant differences in age and implantation period based on gender and environment. However, the implantation age was found to be significantly different between males and females (*p* = 0.028). Furthermore, there were significant differences in all variables tested when comparing the main procedure and the presence/absence of associated diseases. The results suggest that the main procedure and the presence/absence of associated diseases can be significantly impacted by the patient’s age at implantation and the implantation period.

A one-way analysis of variance (ANOVA) was employed to investigate potential disparities among age, implantation age, and implantation duration within the context of four distinct subgroups. In every case, highly significant differences were observed (*p* < 0.001). Given the notable disparities between patients’ quality of life pre- and post-intervention, a correlation analysis was executed to evaluate the association’s strength. This entailed calculating Spearman’s rank correlation coefficient (ρ), implementing a correlation model, and ascertaining a moderately significant correlation (ρ = 0.371, *p* < 0.001).

Upon the study’s completion, a risk analysis was conducted to determine whether congenital bilateral severe-to-profound sensorineural hearing loss (CBSHL) during the initial or subsequent implantation could be deemed a risk factor in the development of otitis. To this end, the odds ratio parameter was calculated, the 95% confidence interval was estimated, and a chi-square test (χ^2^) was applied for statistical significance. Following the analysis, it was deduced that CBSHL constituted a major risk factor for the onset of otitis (OR > 1, *p* < 0.001).

### 3.2. Audiology Assessment

Table 2 presents the results of pre-cochlear implant investigations conducted in the four groups of patients, while the variables analyzed include speech perception, speech production, and reading achievement. Speech perception was assessed using three tests: Word Intelligibility by Picture Identification test (WIPI), Hearing in Noise Test (HINT), and Phonetically Balanced Kindergarten Word List (PBK). The mean accuracy scores for WIPI were only available for the CBSHL2 and NSASHL groups, which were 13.5 ± 9.2% and 20.2 ± 12.7%, respectively. The *p*-value of 0.351 for WIPI suggests no significant difference between these two groups. HINT mean accuracy scores were also only available for CBSHL2 and NSASHL, with values of 27.4 ± 13.8% and 24.9 ± 15.6%, respectively (*p*-value = 0.780), indicating no significant difference between these groups. Finally, PBK mean accuracy scores were available for the CBSHL1 and CP groups, with values of 19.2 ± 14.4% and 22.6 ± 12.9%, respectively. The *p*-value of 0.356 for PBK suggests no significant difference between the CBSHL1 and CP groups in terms of speech perception.

Speech production was assessed using the proportion of correct answers and was applied to the CBSHL2 and NSASHL groups. The mean percentages for correct answers were 33.5% ± 14.2% for CBSHL2 and 38.1% ± 13.6% for NSASHL, with no statistically significant difference between these groups in terms of speech production abilities. Reading achievement was measured as a mean score ± standard deviation and was only applied for the CBSHL2 and NSASHL groups. The mean scores were 74.1 ± 7.6 for CBSHL2 and 71.4 ± 9.2 for NSASHL (*p*-value = 0.555), with no significant difference between these two groups in terms of reading abilities prior to the cochlear implantation.

Table 3 presents the results of the hearing assessment in adult patients at different follow-up time points after cochlear implant rehabilitation. The table consists of four variables, including speech perception (WIPI and HINT), speech production (Correct answers), and reading achievement. The time points are before cochlear implantation and 3, 6, and 12 months after implantation. Regarding speech perception, the WIPI (Word Intelligibility by Picture Identification) test was applied. The mean scores improved significantly from 21.3 ± 11.8% before rehabilitation to 73.4 ± 13.2% at 12 months after rehabilitation (*p* < 0.001). However, there was no significant difference between the 6-month and 12-month time points (*p* = 0.406).

At the HINT (Hearing in Noise Test) assessment, the mean scores also improved significantly from 22.7 ± 13.5% before rehabilitation to 68.4 ± 11.7% at 12 months after rehabilitation (*p* < 0.001). There was no significant difference between the 6-month and 12-month time points (*p* = 0.143). For the speech production tests, the mean percentage of correct answers increased significantly from 33.5 ± 14.2% before rehabilitation to 76.8% ± 19.4% at 12 months after rehabilitation (*p* < 0.001). However, there was no significant difference between the 6-month and 12-month time points (*p* = 0.672). The mean reading achievement scores improved significantly from 76.2 ± 8.1 before rehabilitation to 106.3 ± 19.2 at 12 months after rehabilitation (*p* < 0.001). There was no significant difference between the 6-month and 12-month time points (*p* = 0.737).

### 3.3. Quality of Life Assessment

**Figure 3:**
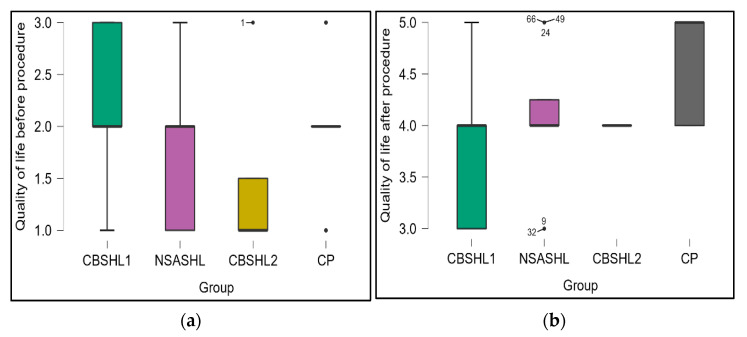
Quality of life assessment using the WHO WHOQOL-BREF (the mental domain). Quality of life reflects the adults’ view and the family’s view about the impact of treatment for their children: $N_{CBSHL1}=20;N_{NSASHL}=12;N_{CBSHL2}=4;N_{CB}=41$; (**a**) quality of life before the procedure; (**b**) quality of life after the procedure; CBSHL1—patients with congenital bilateral sensorineural hearing loss receiving their first implant; NSASHL—patients with non-congenital severe acquired bilateral sensorineural hearing loss; CBSHL2—patients with congenital bilateral sensorineural hearing loss; CP—previously implanted patients who were hospitalized for the change of processor (control group).

**Figure 4:**
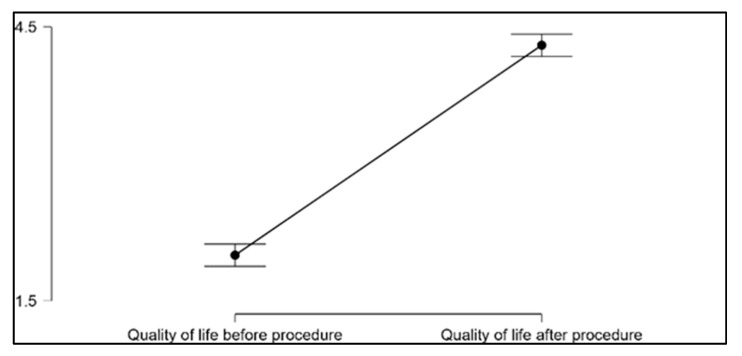
Quality of life: before/after the procedure.

The non-parametric Wilcoxon signed-rank test was used to assess the differences in the mental domain of the WHOQOL-BREF survey before and after cochlear implantation, as this test is particularly suited for small sample sizes and non-normally distributed data. Our results revealed a statistically significant improvement in the quality of life for our patients after undergoing cochlear implantation (*p* < 0.001). The mean quality of life score, as measured by a validated quality of life assessment tool, increased from 2.0 prior to the procedure to 4.2 following cochlear implantation. This substantial increase in the mean score highlights the positive impact that cochlear implantation can have on patients’ overall well-being. The data from this analysis is visually represented in Figure 3 and Figure 4, which provide a clear depiction of the pre- and post-operative quality of life scores for each patient. The figures demonstrate the general trend of improvement in quality of life following the cochlear implantation procedure.

## 4. Discussion

This study represents the first exploration of bilateral cochlear implants conducted in Romania, a developing European nation, offering valuable insights into the advantages and enhancements in the quality of life for adults and children. This study revealed significant differences in age, implantation age, and implantation period among the four groups of patients with varying degrees of sensorineural hearing loss. However, no significant differences were observed in the distribution of place of origin and gender among the study groups. In addition, the pre-cochlear implant investigations reveal no significant differences between the study groups in speech perception, speech production, and reading achievement. The current study also demonstrates significant improvements in speech perception, speech production, and reading achievement for adult patients after cochlear implant rehabilitation. These improvements are evident at the 3-month, 6-month, and 12-month follow-up time points, with no significant differences between the 6-month and 12-month. This data may prove valuable in understanding the characteristics of patients receiving cochlear implants and informing clinical practice and patient management strategies in developing countries with limited funding for cochlear implants.

Recent investigations within the medical literature have identified an increased prevalence of congenital sensorineural hearing loss (CSHL) in male patients, although some studies have demonstrated statistically insignificant disparities between the genders [29]. In the present study, a larger proportion of female patients were observed in comparison to male patients, with 53.25% of subjects being female and 46.75% male. In the current research, the majority of patients with profound congenital sensorineural hearing loss underwent cochlear implant surgery between the ages of 1 and 3 years. The results demonstrated highly significant differences (*p* < 0.001) across all examined variables, indicating that patients with CSHL (Grade 1 or 2) tend to be younger and receive the intervention sooner, with a shorter duration of the procedure.

Several studies have posited that cochlear implantation in children under 12 months of age may yield superior outcomes compared to those implanted after this age, with notable improvements in language acquisition, sound localization, speech, and language development, speech intelligibility, reading comprehension, and auditory perception [8,11,12,13,14]. Speech performance has been found to be significantly correlated with frequency discrimination abilities in cochlear implant users [12], which is crucial for auditory and language acquisition. The age at which implantation occurs is a crucial determinant in the success rate of the procedure, as brain plasticity changes with increasing age [37]. Early implantation confers substantial advantages for children, with improved language acquisition and comprehension observed in those who receive cochlear implants between 1 and 3 years of age [38]. In pre-lingual patients, prompt cochlear implantation is essential for optimizing language and speech rehabilitation outcomes. However, in post-lingual patients, no specific time constraints exist for this intervention [39,40,41].

Additional vital components in the management of cochlear implant patients encompass the interdisciplinary medical team, speech therapist, family, and communication methodologies [42]. Employing gestures and facial expressions is crucial for effectively conveying information to the patient, tailored to their age. Alternative approaches include sign language, pictorial aids, lip-reading, and written communication [43,44]. Despite the substantial improvements in vocal rehabilitation following cochlear implantation in children, limitations persist when compared to their non-hearing-impaired peers. Challenges arise in areas such as information accessibility, communication, social participation, empathizing with others, and academic engagement [17,18]. The significance of family dynamics in influencing the audio-verbal developmental potential of a child with a cochlear implant should not be overlooked [45].

Post-implantation quality of life is generally enhanced for these children, as evidenced by improved language development, communication skills, and speech comprehension. Nevertheless, these improvements are contingent upon several factors, with key determinants being the age at implantation, preoperative language capabilities, duration of implant usage, and communication methods employed during rehabilitation. The medical team, speech therapist, and family play crucial roles in auditory and vocal rehabilitation [46].

Cochlear implantation is a secure surgical intervention for hearing rehabilitation with a low complication rate. Balance disorders, such as dizziness, may manifest due to electrode placement within the inner ear [47]. When opting for cochlear implant surgery in both pediatric and adult populations, individualized treatment approaches should be employed, considering the patient’s age, medical history, and comorbidities. General anesthesia may result in postoperative agitation or drowsiness, necessitating vigilant supervision to prevent falls and subsequent severe injuries or trauma [47].

Minor complications, like otitis, or more severe complications, such as electrode failure, mastoiditis, or facial paralysis, may arise, requiring surgical revision or prolonged hospitalization and corresponding treatments. During the initial months post-implantation, these children are at a higher risk of infection than their normal-hearing counterparts. Otitis can cause significant cochlear or vestibular damage. In our study, it was observed that cochlear implantation might be a significant risk factor for otitis, although age could play a significant role as a confounder, since children are naturally prone to develop otitis, as other studies suggest [48]. Other notable risk factors for infection development include ages below two years and above 65 years. The overall complication rate comprises 14.9% minor complications and 5% major complications, with 42.8% attributed to implant dysfunction. Although important to acknowledge, these complications should not be considered contraindications to cochlear implantation [13].

### Study Limitations

One of the limitations of the current study is that patients were implanted with different devices based on their availability and funding at the time of intervention. Considering the retrospective design of the study, speech perception test scores pre- and post-implant were not evaluated in a standardized manner, although all the included patients underwent audiometry and speech evaluation at the time of hospitalization for implantation. The study may suffer from selection bias if the sample of patients is not representative of the entire population with profound sensorineural hearing loss. Patients who have access to and can afford cochlear implants may have different characteristics from those who cannot. In addition, the study’s sample size is small, and it may lack statistical power to detect significant differences between groups or to draw reliable conclusions about the efficacy of bilateral cochlear implantation. Lastly, including both children and adults, the study may not adequately account for confounding factors that could influence the outcomes, such as age at implantation, the severity of hearing loss, the presence of additional disabilities, or the quality of post-implantation rehabilitation and support.

## 5. Conclusions

After implantation, a significant proportion of patients exhibited improved open-set speech comprehension. Those who had progressive hearing loss, communicated orally during childhood, and used a hearing aid in the implanted ears prior to surgery were more likely to achieve enhanced speech perception outcomes post-surgery. A substantial enhancement in patients’ quality of life was observed after cochlear implantation. Based on the results, adults and children experiencing severe bilateral sensorineural hearing loss should consider cochlear implantation as a viable treatment option. However, personalized treatment approaches should be employed, considering the patient’s age and associated medical conditions. Further governmental funding is needed to provide broad access to cochlear implants.

## Figures and Tables

**Table 1:** General characteristics of the study groups.

Variables	CBSHL1 (*n* = 20)	CBSHL2 (*n* = 4)	NSASHL (*n* = 12)	CP (*n* = 41)	*p*-Value
Place of origin					0.854
Urban	13 (65.0%)	3 (75.0%)	9 (75.0%)	25 (61.0%)	
Rural	7 (35.0%)	1 (25.0%)	3 (25.0%)	16 (39.0%)	
Gender					0.334
Female	7 (35.0%)	2 (50.0%)	8 (66.7%)	24 (58.5%)	
Male	13 (65.0%)	2 (50.0%)	4 (33.3%)	17 (41.5%)	
Age					
Mean	2.1	37.3	17.8	1.9	0.003
Median	2	42.5	15	1	0.059
Mode	1	8	14	1	0.798
Range	3 (4-1)	56 (64-8)	5 (7-2)	50 (60-10)	<0.001
Implantation age					
Mean	1.95	37.33	2.25	4.73	<0.001
Median	1	42.5	2.5	2	0.059
Mode	1	8	3	2	0.272
Range	3 (4-1)	56 (64-8)	2 (3-1)	50 (51-1)	<0.001
Implantation period					
Mean	0.15	0	2.5	13.02	<0.001
Median	0	0	2.5	13	—
Mode	0	0	1	11	0.972
Range	1 (1-0)	0	3 (4-1)	15 (23-8)	0.026
Otitis after CI	15 (75.0%)	2 (50.0%)	3 (25.0%)	10 (24.4%)	0.001

Data represented as *n* (frequency) and compared with Chi-square; CBSHL1—patients with congenital bilateral sensorineural hearing loss receiving their first implant; NSASHL—patients with non-congenital severe acquired bilateral sensorineural hearing loss; CBSHL2—patients with congenital bilateral sensorineural hearing loss; CP—previously implanted patients who were hospitalized for the change of processor (control group).

**Table 2:** Investigations before cochlear implant.

Variables	CBSHL1 (*n* = 20)	CBSHL2 (*n* = 4)	NSASHL (*n* = 12)	CP (*n* = 41)	*p*-Value
Speech perception					
WIPI (mean accuracy% ± SD)	—	13.5 ± 9.2%	20.2 ± 12.7%	—	0.351
HINT (mean accuracy% ± SD)	—	27.4 ± 13.8%	24.9 ± 15.6%	—	0.780
PBK (mean accuracy% ± SD)	19.2 ± 14.4%	—	—	22.6 ± 12.9%	0.356
Speech production					
Correct answers (% ± SD)	—	33.5 ± 14.2%	38.1 ± 13.6%	—	0.570
Reading achievement					
(mean ± SD)	—	74.1 ± 7.6	71.4 ± 9.2	—	0.555

Data represented as *n* (frequency) and compared with Chi-square; CBSHL1—patients with congenital bilateral sensorineural hearing loss receiving their first implant; NSASHL—patients with non-congenital severe acquired bilateral sensorineural hearing loss; CBSHL2—patients with congenital bilateral sensorineural hearing loss; CP—previously implanted patients who were hospitalized for the change of processor (control group); WIPI—Word Intelligibility by Picture Identification test; HINT—Hearing In Noise Test; PBK—Phonetically Balanced Kindergarten Word List.

**Table 3:** Adult patients’ hearing assessment at follow-up.

Variables	Before (*n* = 16)	3 Months (*n* = 16)	6 Months (*n* = 16)	12 Months (*n* = 16)	*p*-Value	*p*-Value *
Speech perception						
WIPI (mean% ± SD)	21.3 ± 11.8%	62.2 ± 16.5%	69.6 ± 12.3%	73.4 ± 13.2%	<0.001	0.406
HINT (mean% ± SD)	22.7 ± 13.5%	55.2 ± 19.1%	61.0 ± 15.8%	68.4 ± 11.7%	<0.001	0.143
Speech production						
Correct answers (% ± SD)	33.5 ± 14.2%	68.1 ± 22.8%	73.5 ± 24.0%	76.8 ± 19.4%	<0.001	0.672
Reading achievement						
(mean ± SD)	76.2 ± 8.1	96.5 ± 12.8	104.1 ± 17.6	106.3 ± 19.2	<0.001	0.737

SD—Standard Deviation; WIPI—Word Intelligibility by Picture Identification test; HINT—Hearing in Noise Test; PBK—Phonetically Balanced Kindergarten Word List; Data analyzed with ANOVA test; * computed *t*-test *p*-value between 6 months and 12 months of rehabilitation after cochlear implant.

## Data Availability

Data available on request.

## References

[1] Carlson M.L. (2020). Cochlear Implantation in Adults. N. Engl. J. Med..

[2] Clinkard D., Barbic S., Amoodi H., Shipp D., Lin V. (2015). The Economic and Societal Benefits of Adult Cochlear Implant Implantation: A Pilot Exploratory Study. Cochlear Implant. Int..

[3] Calháu C.M.D.F., Lima Júnior L.R.P., Reis A.M.D.C.D.S., Capistrano A.K.B., Lima D.D.V.S.P., Calháu A.C.D.F., Rodrigues Júnior F.D.A. (2011). Perfil Etiológico Dos Pacientes Implantados Do Programa de Implante Coclear. Braz. J. Otorhinolaryngol..

[4] Teagle H.F.B., Park L.R., Brown K.D., Zdanski C., Pillsbury H.C. (2019). Pediatric Cochlear Implantation: A Quarter Century in Review. Cochlear Implant. Int..

[5] Beer J., Kronenberger W.G., Pisoni D.B. (2011). Executive Function in Everyday Life: Implications for Young Cochlear Implant Users. Cochlear Implant. Int..

[6] Vahedi S. (2010). World Health Organization Quality-of-Life Scale (WHOQOL-BREF): Analyses of Their Item Response Theory Properties Based on the Graded Responses Model. Iran J. Psychiatry.

[7] De Sousa A.F., Couto M.I.V., Martinho-Carvalho A.C. (2018). Quality of Life and Cochlear Implant: Results in Adults with Postlingual Hearing Loss. Braz. J. Otorhinolaryngol..

[8] Aloqaili Y., Arafat A.S., Almarzoug A., Alalula L.S., Hakami A., Almalki M., Alhuwaimel L. (2019). Knowledge about Cochlear Implantation: A Parental Perspective. Cochlear Implants Int..

[9] Khater A., El-Anwar M. (2017). Methods of Hearing Preservation during Cochlear Implantation. Int. Arch. Otorhinolaryngol..

[10] Vincenti V., Bacciu A., Guida M., Marra F., Bertoldi B., Bacciu S., Pasanisi E. (2014). Pediatric Cochlear Implantation: An Update. Ital. J. Pediatr..

[11] McKinney S. (2017). Cochlear Implantation in Children under 12 Months of Age. Curr. Opin. Otolaryngol. Head Neck Surg..

[12] Firestone G.M., McGuire K., Liang C., Zhang N., Blankenship C.M., Xiang J., Zhang F. (2020). A Preliminary Study of the Effects of Attentive Music Listening on Cochlear Implant Users’ Speech Perception, Quality of Life, and Behavioral and Objective Measures of Frequency Change Detection. Front. Hum. Neurosci..

[13] Craddock L., Cooper H., Riley A., Wright T. (2016). Cochlear Implants for Pre-Lingually Profoundly Deaf Adults. Cochlear Implants Int..

[14] Vieira S.D.S., Bevilacqua M.C., Ferreira N.M.L.A., Dupas G. (2014). Cochlear Implant: The Complexity Involved in the Decision Making Process by the Family. Rev. Lat. Am. Enferm..

[15] Contrera K.J., Choi J.S., Blake C.R., Betz J.F., Niparko J.K. (2014). Rates of Long-Term Cochlear Implant Use in Children. Otol. Neurotol..

[16] Chao T.N., Levi J., O’Reilly R.C. (2018). How Old Is Too Old for Cochlear Implantation for Congenital Bilateral Sensorineural Hearing Loss? Cochlear Implantation Age in Congenital SNHL. Laryngoscope.

[17] Rijke W.J., Vermeulen A.M., Wendrich K., Mylanus E., Langereis M.C., van der Wilt G.J. (2021). Capability of Deaf Children with a Cochlear Implant. Disabil. Rehabil..

[18] Crisan A.F., Oancea C., Timar B., Fira-Mladinescu O., Tudorache V. (2015). Falls, an underestimated risk in COPD. Eur. Respir. J..

[19] Alegre-de la Rosa O.M., Villar-Angulo L.M. (2020). Health-Related Quality of Life in Children Who Use Cochlear Implants or Hearing Aids. Heliyon.

[20] Bianchin G., Tribi L., Formigoni P., Russo C., Polizzi V. (2017). Sequential Pediatric Bilateral Cochlear Implantation: The Effect of Time Interval between Implants. Int. J. Pediatr. Otorhinolaryngol..

[21] Mosnier I., Lahlou G., Flament J., Mathias N., Ferrary E., Sterkers O., Bernardeschi D., Nguyen Y. (2019). Benefits of a Contralateral Routing of Signal Device for Unilateral Naída CI Cochlear Implant Recipients. Eur. Arch. Otorhinolaryngol..

[22] Chang Y.-S., Hong S.H., Kim E.Y., Choi J.E., Chung W.-H., Cho Y.-S., Moon I.J. (2019). Benefit and Predictive Factors for Speech Perception Outcomes in Pediatric Bilateral Cochlear Implant Recipients. Braz. J. Otorhinolaryngol..

[23] Aimoni C., Ciorba A., Hatzopoulos S., Ramacciotti G., Mazzoli M., Bianchini C., Rosignoli M., Skarżyński H., Skarżyński P.H. (2016). Cochlear Implants in Subjects Over Age 65: Quality of Life and Audiological Outcomes. Med. Sci. Monit..

[24] Vieira S.D.S., Dupas G., Chiari B.M. (2018). Repercussões Do Implante Coclear Na Vida Adulta. CoDAS.

[25] Barata P.I., Crisan A.F., Maritescu A., Negrean R.A., Rosca O., Bratosin F., Citu C., Oancea C. (2022). Evaluating Virtual and Inpatient Pulmonary Rehabilitation Programs for Patients with COPD. J. Pers. Med..

[26] Bourn S., Goldstein M.R., Jacob A. (2020). Hearing Preservation in Elderly Cochlear Implant Recipients. Otol. Neurotol..

[27] McKinnon B.J. (2014). Cost Effectiveness of Cochlear Implants. Curr. Opin. Otolaryngol. Head Neck Surg..

[28] Cheng L.-J., Soon S.S., Wu D.B.-C., Ju H., Ng K. (2019). Cost-Effectiveness Analysis of Bilateral Cochlear Implants for Children with Severe-to-Profound Sensorineural Hearing Loss in Both Ears in Singapore. PLoS ONE.

[29] Wachtlin B., Turinsky Y., Herrmann F., Schaefer B. (2017). Phonological Awareness in German-Speaking Preschool Children with Cochlear Implants—3 Case Examples. Int. J. Pediatr. Otorhinolaryngol..

[30] De Raeve L., Archbold S., Lehnhardt-Goriany M., Kemp T. (2020). Prevalence of cochlear implants in Europe: Trend between 2010 and 2016. Cochlear Implant. Int..

[31] Sarah C. (2019). The Strobe guidelines. Saudi J. Anaesth..

[32] Schindler A., Leonardi M., Cavallo M., Ottaviani F., Schindler O. (2003). Comparison between two perception tests in patients with severe and profoundly severe prelingual sensori-neural deafness. Acta Otorhinolaryngol. Ital..

[33] Meyer T.A., Pisoni D.B. (1999). Some computational analyses of the PBK test: Effects of frequency and lexical density on spoken word recognition. Ear Hear..

[34] CADTH (2015). Functional Tests to Assess Speech in Noise. Audiograms and Functional Auditory Testing to Assess Hearing Speech in Noise: A Review of the Clinical Evidence.

[35] Pae H.K., Wise J.C., Cirino P.T., Sevcik R.A., Lovett M.W., Wolf M., Morris R.D. (2005). The woodcock reading mastery test: Impact of normative changes. Assessment.

[36] Wong F.Y., Yang L., Yuen J.W.M., Chang K.K.P., Wong F.K.Y. (2018). Assessing quality of life using WHOQOL-BREF: A cross-sectional study on the association between quality of life and neighborhood environmental satisfaction, and the mediating effect of health-related behaviors. BMC Public Health.

[37] Pereira P.J.S., Souza N.F.H.D., Almeida R.J.D., Menezes D.C., Bom G.C., Trettene A.D.S. (2017). Nursing Diagnoses and Interventions in Children Submitted to Cochlear Implantation. Rev. Esc. Enferm. USP.

[38] Glennon E., Svirsky M.A., Froemke R.C. (2020). Auditory Cortical Plasticity in Cochlear Implant Users. Curr. Opin. Neurobiol..

[39] Fagan M.K. (2015). Cochlear Implantation at 12 Months: Limitations and Benefits for Vocabulary Production. Cochlear Implants Int..

[40] Hsu H.-W., Fang T.-J., Lee L.-A., Tsou Y.-T., Chen S.H., Wu C.-M. (2014). Multidimensional Evaluation of Vocal Quality in Children with Cochlear Implants: A Cross-Sectional, Case-Controlled Study. Clin. Otolaryngol..

[41] Barbosa M.H.D.M., Felix F., Ribeiro M.G., Tomita S., Pinheiro C., Baptista M.M. (2014). Profile of Patients Assessed for Cochlear Implants. Braz. J. Otorhinolaryngol..

[42] Sharma S.D., Cushing S.L., Papsin B.C., Gordon K.A. (2020). Hearing and Speech Benefits of Cochlear Implantation in Children: A Review of the Literature. Int. J. Pediatr. Otorhinolaryngol..

[43] AlSanosi A., Hassan S.M. (2014). The Effect of Age at Cochlear Implantation Outcomes in Saudi Children. Int. J. Pediatr. Otorhinolaryngol..

[44] Necula V., Cosgarea M., Maniu A.A. (2018). Effects of Family Environment Features on Cochlear-Implanted Children. Eur. Arch. Otorhinolaryngol..

[45] Britto F.D.R., Samperiz M.M.F. (2010). Communication Difficulties and Strategies Used by the Nurses and Their Team in Caring for the Hearing Impaired. Einstein São Paulo.

[46] McRackan T.R., Bauschard M., Hatch J.L., Franko-Tobin E., Droghini H.R., Nguyen S.A., Dubno J.R. (2018). Meta-Analysis of Quality-of-Life Improvement after Cochlear Implantation and Associations with Speech Recognition Abilities: QOL Improvement After Cochlear Implantation. Laryngoscope.

[47] Crowson M.G., Semenov Y.R., Tucci D.L., Niparko J.K. (2017). Quality of Life and Cost-Effectiveness of Cochlear Implants: A Narrative Review. Audiol. Neurotol..

[48] Košec A., Živko J., Marković S., Bedeković V., Ries M., Ajduk J. (2022). Impact of preoperative antibiotic use in preventing complications of cochlear implantation surgery. Cochlear Implants Int..

